# Sweden’s excess mortality in 2020–2022 and reporting in the media

**DOI:** 10.1177/14034948241239353

**Published:** 2024-03-18

**Authors:** Martin Lindström

**Affiliations:** Social medicine and Health Policy, Department of Clinical Sciences in Malmö and Centre for Primary Health Care Research, Lund University, Malmö, Sweden

**Keywords:** pandemic, coronavirus, Swedish strategy, excess mortality, mass media, residual confounding, Folkhälsomyndigheten, Public Health Agency, Sweden

## Abstract

**Aim:**

The aim was to scrutinize the report in March 2023 that Sweden’s excess mortality was lowest in 2020–2022 compared with other European Union and Nordic countries, a report that received great national and international attention.

**Study design:**

Comparison of excess mortality in Sweden and Norway.

**Methods:**

Excess mortality for 2020–2022 was calculated for Sweden and Norway, the country with per-capita excess mortality closest to Sweden’s, compared with the average mortality for 2017–2019 in the respective country, following the definitions by *Statistics Sweden* reported in a daily newspaper.

**Results:**

Excess mortality is a measure with low misclassification compared with other pandemic outcome measures. Following the definitions, total excess mortality for the years 2020–2022 was 11,897 individuals in Sweden and 6089 in Norway. However, the distributions of excess mortality across the 3 years strongly differed. In Sweden, 60% of excess mortality was observed in 2020, 8% in 2021 and 32% in 2022. In sharp contrast, 0% of excess mortality was observed in Norway in 2020, 20% in 2021 and 80% in 2022. If the relative distribution of excess mortality in Sweden had been the same as in Norway in 2020–2022, approximately 7000 individuals who died in 2020 would instead have died as excess mortality in 2022, saving approximately 14,000 person-years in Sweden.

**Conclusions:**

**The report disregards residual confounding due to the broad definition of the period 2020–2022. Mass media should avoid one-sided reporting.**

## Introduction

The Swedish COVID-19 strategy has been widely discussed internationally. Until late 2020, the Swedish Public Health Agency (Folkhälsomyndigheten/FHM) was in effect in charge of the efforts issuing recommendations to the 21 regions primarily responsible for the healthcare system, the municipalities responsible for the care of the elderly, other public authorities and the public. The official strategy aimed to reduce the spread to reduce strain on the healthcare system and to protect the elderly and other vulnerable groups. The strategy also aimed to reduce the damage inflicted on important societal functions, reduce the consequences for the public and companies, reduce anxiety and implement correct timely measures [1]. Unofficially, the strategy also included beliefs in the swift achievement of herd immunity [2,3]. In March–June 2020, the per-capita death tolls from COVID-19 in Sweden were ten times higher than in Finland and Norway, and five times higher than Denmark [4].

The high early death tolls triggered the opposition parties to demand a Corona commission scheduled to report before the 2022 election. On 15 December 2020, the first commission report concluded that the strategy had failed to protect the elderly due to the high spread in the population. There had also been a lack of protection equipment, and staffing and organizational problems [5]. The second commission report published on 29 October 2021 and the final report published on 25 February 2022 concluded that the FHM and the government had implemented too late, too few and too weak measures resulting in unacceptably high spread of the virus. Furthermore, the same state authority (FHM) should not be responsible for both public health and infectious disease protection, the government should have included other academic expertise and cooperated with, in particular, the other Nordic countries [6].

The fact that the final Corona commission report was presented on 25 February 2022, the day after the Russian invasion of Ukraine, diverted attention from its critical conclusions. The debate in the parliamentary election in 2022 concerned other issues including energy prices. Newspaper reports in 2022 indicating declining excess mortality in Sweden compared with other countries, when excess mortality for 2021 was added to the initial pandemic year 2020, were highlighted. Still, the reporting that gave rise to most national and international attention was the article in the newspaper *Svenska Dagbladet* published on 4 March 2023, stating that, ‘Sweden’s excess mortality was lowest in the European Union (EU) and the Nordic countries, according to Statistics Sweden’ [7]. It soon became apparent that the newspaper itself had commissioned the calculations by Statistics Sweden [8]. The article asserted that excess mortality had been lower than in any other EU country during the 2020–2022 period [7]. Swedish celebrities responded by praising the initial Swedish strategy [9]. The international reaction was also often praising [10]. There is thus reason to scrutinize these calculations that received such attention, because the wide array of sharp criticism from the commission reports does not harmonize with the notion that Sweden’s initial strategy had been successful.

The objective was to scrutinize the results of the excess mortality calculations for 2020–2022 reported in the newspaper article in terms of systematic error in the study design.

## Methods

Excess mortality for each of the years 2020–2022 was calculated for Sweden and Norway, the country with per-capita excess mortality closest to Sweden’s, compared with the average mortality for 2017–2019 in the respective country. The results from Statistics Sweden reported in the newspaper showed that Norway had 5.0% more deaths in 2020–2022 compared with 2017–2019, and Sweden 4.4%. The use of the average for 2017–2019 and the calculations follow the definitions of excess mortality for the aggregate period 2020–2022 reported in the newspaper.

## Results

**Figure 1.**
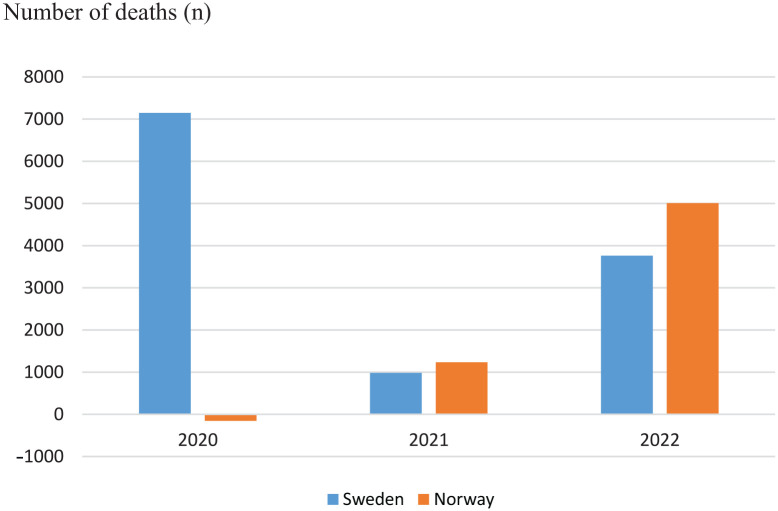
Excess mortality (number of deaths) in Sweden and Norway 2020–2022 compared with average mortality for respective country in 2017–2019.

**Figure 2.**
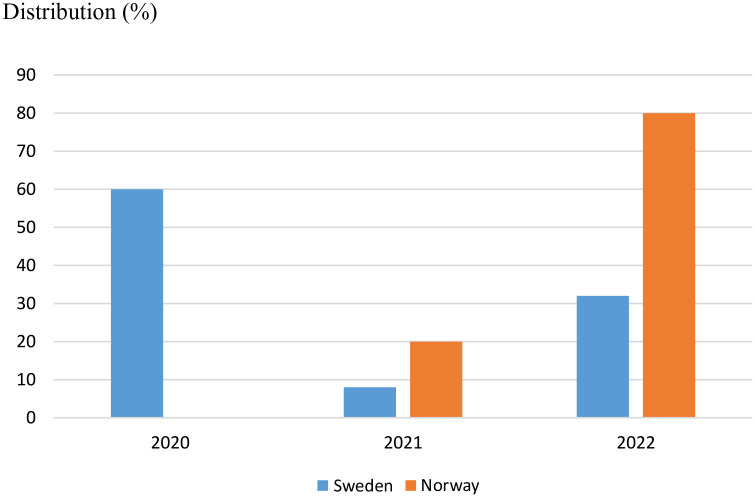
Distribution of excess mortality (%) in Sweden and Norway in 2020–2022 compared with average mortality for respective country in 2017–2019.

Total excess mortality for the years 2020–2022 was 11,897 individuals in Sweden and 6089 in Norway. However, the distributions of excess mortality across the 3 years strongly differed. Figure 1 shows that Sweden’s excess mortality was more than 7000 in 2020, while Norway in fact had lower mortality (−155 individuals) in 2020 than the average for 2017–2019. Instead, excess mortality was almost 5000 individuals in Norway in 2022. Figure 2 shows the relative distribution of the excess mortality in Sweden and Norway. In Sweden, 60% of excess mortality was observed in 2020, 8% in 2021 and 32% in 2022. In sharp contrast, 0% of excess mortality was observed in Norway in 2020, 20% in 2021 and 80% in 2022. If the relative distribution of excess mortality over the years 2020–2022 in Sweden had been the same as in Norway in 2020–2022, approximately 7000 individuals who died in 2020 would instead have died as excess mortality in 2022, saving approximately 14,000 person-years in Sweden.

## Discussion

A total of 60% excess mortality in Sweden occurred in 2020, while 80% of excess mortality in Norway occurred in 2022. The reporting of aggregate excess mortality for 2020–2022 was subject to strong residual confounding due to the broad definition of the period 2020–2022, and the uneven distribution of excess mortality between the two countries across this period. The high number of person-years saved by the time-distribution of excess mortality patterns in Norway was disregarded in the commissioned calculations and in the newspaper reporting, although a major general aim in prevention should be to preserve person-years.

Excess mortality is a valid measure with low misclassification compared with other pandemic outcome measures. Excess mortality has been investigated previously [11], and refined methodologies regarding type of trend used in the calculations, number of years used, impact of pre-pandemic years in an age–sex-specific context, choice of population used for the crisis period (initial versus mean population) and age- and sex-specific patterns of excess mortality have been developed [12]. However, the simple excess mortality measures used in this short communication follow the definitions by *Statistics Sweden* reported by the newspaper, to maximize comparability.

Swedish journalists predominantly supported the initial Swedish strategy. An independent research institute scrutinized the reporting by Swedish mass media during the early part of the pandemic and concluded that Swedish journalists had prioritized ‘informing the population’ instead of asking critical questions [13]. In 2013, political science professor Henrik Ekengren Oscarsson at Göteborgs Universitet suggested the concept ‘opinion corridor’. This concept denotes that if members of a collective, such as journalists, deviate from a rather narrow corridor of opinions they risk losing friends and colleagues, social status and eventually (if they deviate too much) their job. Collective and social control mechanisms, without need of interference from any external actor such as, for example, the government or any other authority, are at work in such processes [14]. The notion that a consensus culture existed is supported by the fact that the Swedish state authority Myndigheten för Samhällsskydd och Beredskap even criticized the free mass media for not asking critical questions during the first pandemic year 2020 in one of its own reports [15].

Why was excess mortality in Norway (and other countries) higher in 2021–2022? First, many elderly and weak individuals died in Sweden in 2020 due to the initial strategy. The pattern in Norway may thus have an autoregressive component following the fact that many lives in this group were saved in 2020 compared with Sweden. Second, parts of the pandemic handling under the new vaccination regime may have been more effective in Sweden in 2021–2022 than in other countries. This second point should be investigated instead of disregarding the shortcomings of the initial Swedish strategy with calculations burdened by substantial residual confounding due to the definition of broad periods for analysis. Such news media reporting of 3-year-period excess mortality may serve to mislead major parts of the public who will not consider the huge differences in year-by-year excess mortality between Sweden and, for example, other Nordic countries. In effect, such reporting will prevent a sound public debate concerning the early Swedish strategy and constructive suggestions to amend the shortcomings.

## Conclusion

In Sweden, 60% of excess mortality was observed in 2020. In sharp contrast, 0% of excess mortality was observed in Norway in 2020 and 80% in 2022. The newspaper reporting was thus subject to residual confounding following the broad definition of the period 2020–2022. Mass media should avoid one-sided reporting.
